# Development and Validation of a Family Education Program for Persons with Dementia in India

**DOI:** 10.1002/alz.095239

**Published:** 2025-01-09

**Authors:** Kshama Bangera, Sebestina Anita Dsouza

**Affiliations:** ^1^ Manipal Academy of Higher Education, Manipal, Karnataka India

## Abstract

**Background:**

In low and middle‐income countries like India, with limited treatment and support options, persons with dementia are primarily looked after by their families. Dementia’s impact extends beyond the primary caregiver to the entire family. Involving and supporting family members in care can enhance the primary caregiver’s well‐being and improve the quality of care for the person with dementia. While family‐based approaches have been studied in Western countries, their applicability may be limited in collective cultures like India. Currently, there are no suitable educational interventions tailored to the Indian context. This study aims to fill this gap by developing and validating a education program for families of persons with dementia in India.

**Method:**

The family education program, developed based on existing literature and WHO guidelines, was tailored for the Indian context. Primary caregivers and one family member were required to attend four sessions, which included PowerPoint presentations, a handbook, and activities. A total of 15 stakeholders, including service providers (occupational therapists, social workers, psychiatrists, psychologists) and service users (primary caregivers and family members), were purposively selected for validation. The education program was shared with stakeholders, and their feedback was collected through semi‐structured interviews. Their suggestions were analyzed and integrated into the program, which was subsequently shared again for additional feedback.

**Result:**

Analysis of the interviews suggests the stakeholders appreciation for educating other family members besides the primary caregiver and found the content relevant to the program’s objective. They suggested strategies to improve the program’s acceptability, such as simplifying the language, reorganising and reducing the content, using more interactive activities, and tailoring the program for the families. Reducing program duration for better feasibility and acceptance was also recommended. These suggestions were incorporated, and a case‐based approach was introduced to facilitate delivery. The stakeholders were satisfied with the modifications, especially the case‐based approach, but raised concerns about the program’s duration and suggested flexibility based on family needs during delivery.

**Conclusion:**

The family education program for dementia patients was deemed contextually relevant and valid. A pilot test will assess the program’s acceptability and feasibility in supporting caregivers of people with dementia.

